# Towards intelligent complex networks: the space and prediction of information walks

**DOI:** 10.1007/s41109-019-0140-5

**Published:** 2019-06-13

**Authors:** Chuankai An, A. James O’Malley, Daniel N. Rockmore

**Affiliations:** 1 Department of Computer Science, Dartmouth College, Hanover, 03755 NH USA; 2 Department of Biomedical Data Science and the Dartmouth Institute of Health Policy and Clinical Practice in the Geisel School of Medicine at Dartmouth College, Lebanon, 03784 NH USA; 3 Department of Mathematics, Dartmouth College, Hanover, 03755 NH USA; 4 External Faculty, The Santa Fe Institute, Santa Fe, NM, 87501 USA

**Keywords:** Information walk prediction, Network measures, Bayesian personalized ranking, Patient referral network, Outlier detection

## Abstract

In this paper we study the problem of walk-specific information spread in directed complex social networks. Classical models usually analyze the “explosive” spread of information on social networks (e.g., Twitter) – a broadcast or epidemiological model focusing on the dynamics of a given source node “infecting” multiple targets. Less studied, but of equal importance is the case of *single-track* information flow, wherein the focus is on the node-by-node (and not necessarily a newly visited node) trajectory of information transfer. An important and motivating example is the sequence of physicians visited by a given patient over a presumed course of treatment or health event. This is the so-called *a referral sequence* which manifests as a path in a network of physicians. In this case the patient (and her health record) is a source of “information" from one physician to the next. With this motivation in mind we build a Bayesian Personalized Ranking (BPR) model to predict the next node on a walk of a given network navigator using network science features. The problem is related to but different from the well-investigated link prediction problem. We present experiments on a dataset of several million nodes derived from several years of U.S. patient referral records, showing that the application of network science measures in the BPR framework boosts hit-rate and mean percentile rank for the task of next-node prediction. We then move beyond the simple information walk to consider the derived network space of all information walks within a period, in which a node represents an information walk and two information walks are connected if have nodes in common from the original (social) network. To evaluate the utility of such a network of information walks, we simulate outliers of information walks and distinguish them with the other normal information walks, using five distance metrics for the derived feature vectors between two information walks. The experimental results of such a proof-of-concept application shows the utility of the derived information walk network for the outlier monitoring of information flow on an intelligent network.

## Introduction

It is common to represent interactions between people using a (social) network, which – regardless of the defining notion of connection – could produce a mathematical structure reflecting the possible paths for the flow of information between the actors. The person-to-person communication in such a network turns into a *path* (Wikipedia), or more accurately a *walk* (Wikipedia), since it is possible (and in many contexts even likely) for the “walker" (e.g., news) to revisit some person (node)^1^. Indeed, multiple “visits" can provide a kind of reinforcement of the information of interest that might be relevant to its learning or absorption. This node-by-node (e.g., person-after-person) information spread model – a “single-track" model – is a kind of epidemiological model but different from the classical diffusion/broadcasting models that are often used in the analysis of social media. A single-track model of spreading will not produce the kinds of exponential growth (of “infected nodes”) in each round.

Single-track information spread is appropriate to our particular interest: the problem of *next visit prediction* of a walker in a network. Our original motivation arises from research on physician collaboration networks built by referrals (An et al. 2018a), where two nodes/physicians are directly connected with a weighted edge if they have been visited by the same patients within a given time period. Patients “walk" this network in the course of a presumptive treatment event. A predictive application based on the features of such “referral sequences" (a given “visit" is enabled by the first physician making a referral for the patient to the second physician) may provide a better understanding of the process of collaboration among health professionals. Furthermore, precise prediction of the next visited physician may help with the efficient allocation of medical resources for a patient’s treatment. Other examples of single-track information walks in different contexts include a traveler visiting preferred places, consumers traversing stores in a shopping mall, or the work history of an employee. In all these cases, the records of transitions define the corresponding network, where the existence and times of visiting some nodes in history will influence the future possibility of visiting (transition) between a pair of nodes. Indeed, a walker may be the first to ever traverse from one node to another – suggesting that these nodes did not connect each other in history records. Therefore, a more accurate framing is the problem of *visit prediction* for a walker in a *state space*. Since we assume a context of information sharing and metadata in specific domains, we name these *information walks* and the problem in our research as *information walk prediction*. In the above instances the entire walk up to the last node may directly affect the selection of the next visited node, so that this problem is generally not a memoryless Markov chain.

In previous work we analyzed a network of physicians (see An et al. (2018a, c)) based on U.S. patient referral records, which pave the way for research on information walks. Herein, exploiting both metrics proposed in our analysis (An et al. 2018a, c) and classical network science measures, we propose a numerical score to model the preference/attractiveness between the last observed node on an information walk and any possible candidate node in the network. This score takes multiple feature vectors from the targeted information walk as well as several groups of involved nodes. Based on the preference score, we apply a general Bayesian Personalized Ranking (BPR) framework to represent the goal of next-node prediction in an objective function so that the problem could be solved by machine learning. We use a large U.S. patient referral network as an important real-world setting for applying our methodology. Several network science measures (e.g., node centrality) in the national physician network facilitate the prediction for a pair of nodes including those not directly linked in the past. The study and comparison of several models demonstrate that features of an information walk improve the BPR framework since it exceeds Long Short-Term Memory (LSTM) and other metrics reflecting standards used in link prediction research. The main reasons for such an improvement is the inclusion of network science measures with other metadata features.

This paper moves beyond our published paper (An et al. 2018b) in the Complex Networks 2018 conference through its introduction and use of the relationships among multiple ongoing information walks. We also investigate the space of information walks with a network science model, in which each node represents an information walk and an edge connects two nodes of information walks if they share at least one common node (e.g., the same physician) in the originating network. We find several significant patterns in the new network of information walks and verify them via a statistical test.

A key contribution is our identification of criteria to label an information walk with different structural patterns in the network of information walks as an outlier. We use a simulation-based test of information walk outliers in the network of information walks in order to (1) demonstrate the efficacy of the model for the information walks network; (2) complement the proposed BPR-IW model of walk prediction since the users of an intelligent network platform may not have time to focus on every walk and check the prediction of its future direction while an overall outlier detection function could filter some “abnormal” or “new” walks and remind users to check. In related work (Savage et al. 2014; Ranshous et al. 2015; Eswaran and Faloutsos 2018; Takahashi et al. 2011), researchers have targeted different parts in a graph to build a specific outlier detection algorithm, including nodes, subgraphs, separate point-to-point edges (e.g., TCP-IP communication, connections between new accounts in social networks). Herein we are the first to implement outlier detection for a whole information walk, which differs from prior work due to the existence of the same single “walker” or information flow along the sequence of visited nodes.

We simulate the outlier information walks with random replacement of their nodes, explore the measures of an information walk in a network of information walks, and design five distance metrics (based on the walk features) within a general outlier detection framework to distinguish the simulated (outlier) information walks from those actually observed. Moreover, since an outlier information walk may be an abnormal or creative (e.g., new treatment procedure) case, the initial results suggest a way to contribute to a more intelligent network via outlier detection for ongoing information walks, which complements our proposed BPR walk prediction model.

“Related works” section surveys related works about information diffusion and outlier detection in a network. In “Proposed models” section, we introduce the BPR framework with a preference score for the task of information walk prediction, and detail the construction of a network of information walks and the model of outlier detection for information walks. “Evaluation of walk prediction” section provides the details of our walk prediction experiment. “Patterns in network of information walks” section analyzes the network of information walks and reports key patterns within it. “Simulation test of outlier detection” section shows the result of a simulation test of information walk outlier detection. Finally, “Conclusions” section summarizes our findings and suggests directions of further research.

## Related works

The focus of our work is related to but different from the well-investigated problem of *link prediction*, in which given a dynamic network observed at a time point, (possibly new) links in the future are predicted (e.g., Martínez et al. (2017)). Another related problem is finding *missing links* (Nakagawa and Shaw 2004). Traditional link prediction models (e.g., Adamic and Adar (2003) and Liben-Nowell and Kleinberg (2007)) usually only rely on a form of node similarity derived from network topology and generally ignore the whole (information) walk. Many past works target the problem of multitrack spreading or broadcasting in a *directed acyclic graphs* (DAGs), while our proposed information walk model allows the existence of a loop. Representative works include the Independent Cascade (IC) model (Kimura and Saito (2006) and Bourigault et al. (2014)), the Linear Threshold (LT) model (He et al. 2012), and probabilistic methods (Gomez-Rodriguez et al. 2011; Myers and Leskovec 2010). In addition, past works do not consider observed information walks as a part of their key inputs. In contrast, we incorporate information walks using summary measures of network features in the corresponding network. Diffusion models are clearly different, and they have been introduced to the research of epidemics (Raj et al. 2012).

In other domains, several applications (most notably online shopping or search) try to predict a visit to a next “item”. The general BPR model (Rendle et al. 2009) previously has been introduced to online shopping (Rendle et al. 2010) to serve users with personalized goods recommendations. A common problem has been to predict the next place of work of a given employee in a labor pool using LSTM (Li et al. 2017) or a “gravity law" (James et al. 2018) based approach. Choi et al. (2016) applied deep learning to estimate the next medication code in a course of treatment by combining codes of medical treatment and physician visiting records to obtain a comprehensive feature representation.

The idea of state transition (e.g., Leibon and Rockmore (2013)) has been tested for an accurate recommendation. Recently, a Transition-based Factorization Machine model (TFM) (see He et al. (2017) and Pasricha and McAuley (2018)) was used to predict the next state in an abstract space of items for users. In contrast to the TFM model, our proposed preference score model considers network science measures and shows the benefits of incorporating them with other metadata features (more details can be found in “Evaluation of walk prediction” section).

Outlier detection (i.e., anomaly detection) is a thoroughly investigated problem in the field of applied machine learning. A survey paper (Ranshous et al. 2015) reviewed diverse methods of outlier detection in a graph from multiple perspectives, including nodes, edges, subgraphs, and changes due to an event. Another survey (Gupta et al. 2014) applied outlier detection methods to temporal data. Recently, researchers targeted the outlier “bridge edges" in a streaming of graphs (Eswaran et al. 2018; Aggarwal et al. 2011) or a streaming of separate point-to-point edges (Eswaran and Faloutsos 2018; Takahashi et al. 2011). Our proposed outlier detection method deals with the new target of information walks, which considers both overall structural and temporal patterns. Past work on scan statistics (Park et al. 2009) for a single abnormal edge detection has focused on predicting local patterns at every step. In contrast, we target the whole information walk and use time series features derived from a network of information walks to inform outlier detection.

There is an extensive statistical literature (Barnett and Lewis 1974; Hido et al. 2011; Hodge and Austin 2004) on outlier detection, including that for longitudinal data. In statistical methodology and applications, outlier detection is often characterized by the study of residuals or other measure of deviation of the estimated or fitted values of a model from the observed (i.e., true) values. For example, if the data are assumed to follow a normal distribution law, one might compute the number of residual standard deviations away from the mean in order to rank the observed residuals from most to least indicative of an outlier. In non-parametric statistical models, a statistic for measuring the distance of an observation from the value most expected in the absence of outliers is specified from the onset, as opposed to being implied by the modeling assumptions. We propose new distance metrics to be maximized by a proximity based outlier detection algorithm. As this is the first paper to introduce an information walk network, we assess our outlier detection methods for the purpose of demonstrating the utility of an information walk network rather than seeking to find the optimal outlier detection method among several options (e.g., algorithmic and statistical models). This later task may be undertaken once the concept of an information walk network has been proven.

## Proposed models

In this section, we begin with a preference model for information walk prediction, then describe how to build a network of all information walks, and a proximity-based unsupervised framework for information walk outlier detection.

Given an observed information walk in a directed network, the first task is to predict the next visited node. To do so we build a numerical preference/attraction score for the observed part of an information walk (including the last node visited and an overall feature comprising all past visited nodes) and any possible next-visited candidate. Therefore, when predicting which node would be more likely to be visited by a walker, we can compute and sort the preference/attraction scores over all candidate nodes. We then pick out a small number which have a comparatively large score. As a result, this prediction framework allows for the convenient detection of possible choices from the returned list (see Fig. 2 for an illustration of the identification process). “Results” section evaluates the performance of the prediction in terms of a returned candidate list. The definition of a preference score is a key component of the algorithm.

To formalize the problem, let *P* denote the set of all chronological node sequences (i.e., information walks). For an information walk *i*∈*P*, *p*_*i*_ represents the feature vector of the observed sequence of nodes at a time point *T*, *c*_*i*_ refers to the last node on information walk *i* before time point *T*, *f*_*i*_ is the first node on information walk *i* after *T* (i.e., the actual next visited node). Let *J* represent the set of possible candidates, which could cover a wide range of nodes, even the whole network except *c*_*i*_, or just a subset of nodes in the network after filtering to speed up the computation if the network is large. *X*(*p*_*i*_,*c*_*i*_,*j*) denote the preference/attraction score between the last observed node *c*_*i*_, the overall walk feature *p*_*i*_ and a candidate *j*∈*J* for the next node. We aim to derive an objective function and train the preference-related parameters to make *X*(*p*_*i*_,*c*_*i*_,*f*_*i*_)>*X*(*p*_*i*_,*c*_*i*_,*j*) for as many candidates *j*∈*J* (and *j*≠*c*_*i*_) as possible. If so, it indicates that a model predicts the next node on an information walk (i.e., the future direction in a network space) more accurately.

Diverse groups of network science features, either exogenous (metadata) or endogenous (topological) about the observed walk, may boost the accuracy of information walk prediction. Our published papers (An et al. 2018a, c) offer groups of such features useful for building our new preference score model. Table 3 shows a detailed list of features used in prior walk-prediction analyses.

### Preference/Attraction score

We define a preference score *X*(*p*_*i*_,*c*_*i*_,*j*) in the BPR framework, called *BPR-IW* using the feature vector *p*_*i*_ of the information walk. The other factors in the preference/attraction score are the last/current node *c*_*i*_ of walk *i* and a node *j*∈*J* as the candidate:

1 $$  X(p_{i}, c_{i}, j) = p_{i}^{T}V\beta_{c_{i}}+p_{i}^{T}S \gamma_{j}+\beta_{c_{i}}^{T}U\gamma_{j}+wd(c_{i},j)  $$

where in Eq. 1 the superscript ^*T*^ refers to the transpose operator for a matrix. *p*_*i*_ means the overall feature of the whole observed part of information walk *i*. $\beta _{c_{i}}, \gamma _{j}$ represent the feature vector of the last node *c*_*i*_ and a candidate node *j*, respectively. *d*(*c*_*i*_,*j*) represents the distance between *c*_*i*_ and *j* in terms of their profile similarity based on the metadata. Three matrices *V*,*S*,*U* about the node-walk interactions will be trained as model parameters, which represent both all possible interactions in real life and the feature interactions that exist in a theoretical Factorization Machine (Rendle 2010) or Polynomial Regression (Theil 1992) model. In addition, another parameter *w* (weights) adjusts the importance of node profile similarity, which corresponds to the last group of features in Table 3. To make the matrix operation in Eq. 1 clear, Table 1 shows the dimension of several key parameters/vectors.

**Table 1:** Dimension of the model parameters/features in Eq. 1

Feature	Dimension	Note	Parameter	Dimension	Note
*p*	*M*×1	Information walk	*V*	*M*×*N*	Walk-node interaction
*β*	*N*×1	Last node	*S*	*M*×*H*	Walk-node interaction
*f*,*γ*	*H*×1	Ground truth / candidate	*U*	*N*×*H*	Node interaction
*d*	*L*×1	Profile similarity	*w*	1×*L*	Profile weight

Equation 1 takes multiple factors into consideration when predicting the next visited node on an information walk. *S*,*V* represent the interaction between the initial part of the walk and the candidate/ground truth node, respectively, while *U* describes the extent of matching between the candidate and the last node on the walk which might influence the decision of the future direction. Network science provides the widely applicable features *p*,*β*,*f*,*γ*, since they can be computed from the topological structure of a network, regardless of the type of metadata in the network. As the profile distance *d* relies on the context (e.g., physician specialty), we distinguish it from the other features.

### Learning BPR-IW model

Equation 1 defines a preference score *X*(*p*_*i*_,*c*_*i*_,*j*) for sorting candidate nodes in *J* for an information walk *i*. When evaluating the ranking of candidate nodes for an information walk, it is convenient to get the scores for all candidates, and then pick the top-K candidates. In this way, the relative order of the score counts more than the actual values. The Bayesian Personalized Ranking (BPR) framework (Rendle et al. 2009) defines the objective function as finding the optimal fitting MAP estimator with the use of regularization to guide the choice of predictors. The crucial part of this Bayesian procedure is the evaluation of the posterior probability of the model parameters conditional on the network (i.e., the interactions among nodes stemming from patients’ preferences about the next physician they visit). The procedure is presented mathematically in Eq. 2:

2 $$  \Theta = arg \underset{\Theta}{max} \sum_{i \in P_{train}} \sum_{j \in J\setminus\{c_{i},f_{i}\}} log \sigma(\hat{X}(p_{i}, c_{i}, f_{i}) - \hat{X}(p_{i}, c_{i}, j)) - \frac{\lambda_{\Theta} }{2}||\Theta||^{2}  $$

where *σ* represents the sigmoid function *σ*(*x*)=(1+ exp(−*x*))^−1^. Using the sigmoid function, the gap between two preference scores for two candidate nodes is mapped into the interval (0,1) so that the loss function is defined even if the gap diverges to infinity when computing the optimal model parameters. The components of *σ*, $\hat {X}(p_{i}, c_{i}, f_{i}) - \hat {X}(p_{i}, c_{i}, j)$, describe the gap in the preference scores between the ground truth of the current walk, *f*_*i*_, and another possible candidate, *j*. *P*_*train*_ refers to the training set information walks. In the objective function (2), *Θ* is a general set parameter to be learned in the training process, such as *V*,*S*,*U*,*w* introduced by Eq. 1. We can use several random matrices/vectors drawn from a multivariate Gaussian distribution as initial values. The values of the model parameters will be optimized in the iterative training process. As the last item, *λ*_*Θ*_ regularizes the objective function to avoid overfitting.

According to the size of the dataset in Table 2, the number of pairs of information walks and candidate nodes *O*(|*P*_*train*_||*J*|) is huge (more than 1 billion). In this case, stochastic gradient descent (SGD) optimizes Eq. 2 efficiently, which updates the set of parameters *Θ* based on the derived gradient in Eq. 3. To update the parameters in each round of SGD with an information walk *i* and a candidate node *j*, the gradients of Eq. 2 for a parameter *θ*∈*Θ* are:

3 $$  \begin{array}{lcl} \frac{\partial}{\partial \theta}\left(log \sigma\left(\hat{X}(p_{i}, c_{i}, f_{i}\right) - \hat{X}(p_{i}, c_{i}, j)) - \frac{\lambda_{\theta}}{2}||\theta||^{2}\right)\\ = (1 - \sigma\left(\hat{X}(p_{i}, c_{i}, f_{i}) - \hat{X}(p_{i}, c_{i}, j)\right)\frac{\partial}{\partial \theta}\left(\hat{X}(p_{i}, c_{i}, f_{i}) - \hat{X}(p_{i}, c_{i}, j)\right) - \lambda_{\theta}\theta \end{array}  $$

**Table 2:** Size of training, test and candidate sets at different time points in 2011, which are derived from the TDI dataset

Date of observation *T*	*P*_*train*_	*P*_*test*_	Candidate nodes *J*
03/01	17.6K	18.7K	16.6K
05/01	51.8K	19.5K	17.3K
07/01	83.7K	16.6K	14.9K
09/01	113.1K	15.8K	14.3K
11/01	142.4K	16.8K	15.1K

**Table 3:** Features about information walks and related nodes including applicable network measures, new metrics defined by our past analysis (An et al. 2018a), and a few from the metadata of medical treatment records, such as Relative Value Units (RVU) of medical service

Group	Measures
Information walk *p*	Number of nodes on it, time range, pairs of mutually connected nodes, sum of RVU for all visiting, number of visited hospitals, average node PageRank values
Next node/candidates (*j* and *f*)	Clustering coefficient, PageRank, Hindex, number of initiated cross hospital referral region referrals
Last node *c*	Beyond the features in the group of next node/candidate: time gap with last occurrence, RVU, a binary flag of multiple occurrences on the walk, a binary flag of working in the same hospital previous physician (node)
Metadata for profile similarity *d*(*c*,*j*)	Indicators of the same specialty/residency hospital/hospital referral region, number of referrals in history.

Our paper (An et al. 2018b) shows the full list of applied measures

The partial derivative of $\hat {X}(p_{i}, c_{i}, f_{i}) - \hat {X}(p_{i}, c_{i}, j)$ with respect to some parameter could be computed by Eq. 1. Equation 4 gives the instances of *S* and *U* that are defined in Table 1. Note that due to an offset in the gap of two preference scores, it is not necessary to update *V*.

4 $$  \begin{array}{lcl} \frac{\partial}{\partial S} (\hat{X}(p_{i}, c_{i}, f_{i}) - \hat{X}(p_{i}, c_{i}, j))= p_{i} \gamma_{f_{i}}^{T} - p_{i} \gamma_{j}^{T} \\ \frac{\partial}{\partial U} (\hat{X}(p_{i}, c_{i}, f_{i}) - \hat{X}(p_{i}, c_{i}, j))= \beta_{c_{i}}\gamma_{f_{i}}^{T} - \beta_{c_{i}}\gamma_{j}^{T} \end{array}  $$

### Network of information walks

An information walk not only transmits information across nodes, but also connects the neighboring nodes in the space of all information walks. Here we define the network of information walks to model the space of all information walks, in which a node represents an information walk and two nodes are connected when they share at least one node in the originating social network.

**Fig. 1:**
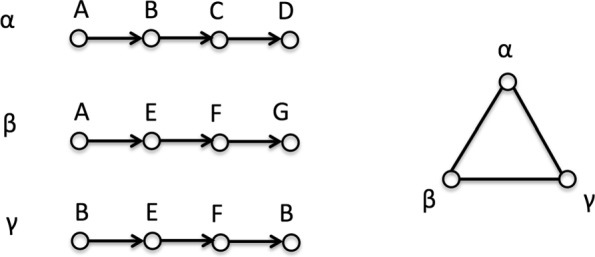
Three information walks with nodes from A, B... G, and the corresponding network of information walks

In the network of information walks, several edge weights distinguish the relationship between two connected nodes (i.e., information walks), such as the number of distinct common nodes, the Jaccard index (Jaccard 1901) (size of intersection divided by size of union) of two sets of originating nodes on two information walks. Figure 1 shows an example of the network of information walks. Here *α*,*β*,*γ* are three information walks with several nodes (*A*,*B*,…,*G*). Since every pair of information walks share at least one node, the corresponding information walk network is an undirected 3-node clique. For the edge linking *β* and *γ*, the number of common nodes is 2 (nodes *E* and *F*), and the Jaccard index weight is 2/5=0.4 because in total there are five kinds of nodes on them.

**Fig. 2:**
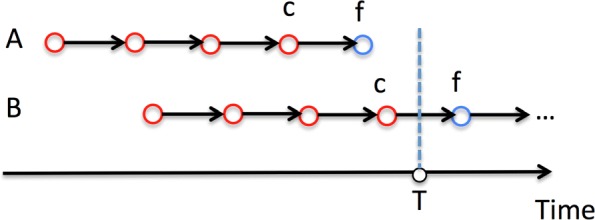
At a given time point *T*, two information walks (*A* and *B*) belong to the training and test set, respectively

The network of information walks should be very dense. In the originating social network, node degree refers to the number of directly linked neighbors, but in the network of information walks, it corresponds to the number of nodes (information walks) that are connected by the original node, and sets a lower bound of the size of a clique, since other nodes in the originating social network may extend the clique.

### Outlier detection for information walks

As a complimentary task for walk prediction, the outlier detection of information walks identifies the information walks that deviate from the expected topological structures in an unsupervised set of information walks via the features derived from the network of information walks. The target of outlier detection is the whole information walk rather than a single node or edge on it. We hope that such a detector could provide an early stage “alert", identifying “abnormal” or favorable novel information walks to improve the safety of subsequent carried information and the robustness of the network. To implement outlier detection, we need to define key features of the network of information walks and use these to design an algorithmic outlier detector. 
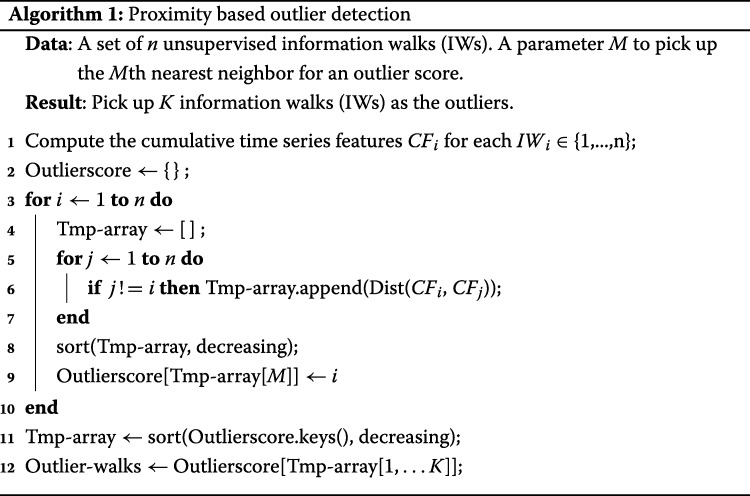


An information walk expands node by node. Thus the evolution of an information walk could be presented by a series of cumulative feature vectors at each timestamp when the walker visits a new node. The full list of applied features will be introduced in “Simulation test of outlier detection” section. We present the general algorithmic outlier detection framework in Algorithm 1, which requires a distance metric function between any pair of information walks.

Algorithm 1 is an unsupervised proximity-based outlier detection framework. The key idea is to compute an “outlier-score" for each IW to pick the *K* information walks with the largest *K* scores. The data preparation step refers to Line 1 in Algorithm 1, where we compute the time series features for every information walk. In Lines 2-10, we compute the pairwise distance between two **information walks** with some metric function (introduced later in this subsection) and treat the distance to the *M*th nearest neighbor as the outlier score. Finally, in Lines 11-12, we sort the outlier score to get the Top-*K* candidates of outliers. Under a time complexity of *O*(*n*^2^), Algorithm 1 more easily adapts to diverse kinds of proximity measures than statistical outlier detection methods that are reliant on assuming probability distributions of the residuals and models the degree to which IW is an outlier. A drawback is that the algorithm might be sensitive to the choice of *M* when defining the outlier score, making it necessary to tune the parameter *M* for each experiment.

Assume we extract *P* different measures of an ongoing information walk at *T* timeslots on the time axis. In total, the feature vector is then a *P*×*T* tensor. An equal-weighted distance function sums up the distance of each measure. Therefore, the *Dist* function in Line 6 could be transformed to a distance function between a pair of numerical arrays of each measure, but their lengths may be different due to the varying lengths of information walks. Denote the longer array as *LA* and the shorter one as *SA* and their lengths as *l* and *s*, respectively. Here we propose or apply five distance metrics to complete Algorithm 1.

- Sliding substring matching (SSM). To match the shorter array *SA*, enumerate all *s*-length consecutive subarrays from *LA* and take the minimum Manhattan Distance between a subarray in *LA* and the *SA*.
- Edit distance/Dynamic Time Wrapping (ED/DTW). Equation 5 describes the state transition equation for the dynamic programming model, in which *d*(*i*,*j*) is the distance between the first *i* units in *LA* and the first *j* units in *SA*. The initial settings are *d*(*i*,0)=*i*×*λ* for *i*∈[1,*l*]*d*(0,*j*)=*j*×*λ* for *j*∈[1,*s*]. *λ* is the penalty factor to represent the cost of skipping a unit in an array. After the process of dynamic programming in Eq. 5, the value of *d*(*l*,*s*) is our desired distance.

5 $$ d(i,j) = min \left\{\begin{array}{ll} d(i-1, j-1) + abs(LA[i] - SA[j]), \\ d(i, j-1) + \lambda, \\ d(i-1, j) + \lambda \end{array}\right.   $$

- Interpolation. Treats *LA* and *SA* as several discrete samples from a function of time in the interval [0, 1], in which the first unit in *LA* and *SA* is at zero while the last unit is at one. The rest of the non-extreme units are allocated with an equal interval. For example, if *LA*=[0.1,0,2,0.3,0,4,0.5], the corresponding time intervals would be (0,0.1),(0.25,0.2),(0.5,0.3),(0.75,0.4),(1.0,0.5). To align *SA* and *LA* we take the simple linear interpolation for the corresponding points of *LA* to get new points that have the same time-index with *SA*. Finally, we compute the pairwise Manhattan Distance.
- Longest common substring (LCS). The LCS method originally aims to find the longest subsequence common to two strings. In contrast to substrings, subsequences are not required to occupy consecutive positions within the original sequence. Two numerical units are treated as equal if their abstract distance is less than the threshold.
- Sliding substring averaging (SSA). Starting from the first node in *LA*, set a sliding window of length of *l*−*s*+1 and extract the average of those units in *LA* covered by the sliding window. The sliding window moves right one unit each iteration to generate *s* values from *LA*, so that it is able to compute the distance between the derived values and *SA*.

## Evaluation of walk prediction

### Dataset

The data for our analyses are the U.S. Medicare beneficiary insurance claims for a subgroup of patients over 2007–2011. These data, obtained via a data use agreement by TDI^2^, contain the patient-physician visiting records for patients who suffered from cardiovascular disease. With this information we are able to link physicians according to patient ID. A *referral* is defined as the event in which two physicians are visited by the same patients within a short time interval. We then derive a *sequence of referrals* (An et al. 2018a) for the same patient, which corresponds to an information walk in the professional network of physicians. Because a referral sequence may have loops (e.g., *A* to *B* to *A*for two physicians), some nodes may be revisited. Therefore, the referral sequence corresponds to an information walk on the physician network rather than a “path” without repeated nodes.

For the set of information walks *P* in a year, given an observation time point *T* we build the training set *P*_*train*_ to store the walks ending before *T*. The test set *P*_*test*_ includes the walks that are ongoing at *T*. Figure 2 illustrates two examples. Since information walk *A* terminates before time point *T*, it is in the training set *P*_*train*_. At the time point *T*, a node on walk *B* is still passing information to the next node, so walk *B* belongs to the test set *P*_*test*_. In *A* and *B*, the observed red nodes contribute to the overall information walk feature *p*. For a walk in *P*_*train*_, all nodes but the last one belong to the observed part, while the last node serves as the ground truth *f*. The candidate set *J* contains the ground truth *f* of all walks in *P*_*test*_; thus it randomly samples a subset of nodes in the whole network.

The U.S. physician collaboration network derived from the TDI dataset provided 4.66M information (referral) walks in 2011. The training and test set are defined as information walks with at least six visits. Table 2 presents the size of training and test sets at several time-points *T*, as wells as the candidate node set *J*. The size of *P*_*train*_ increases from March to November, since it contains all information walks ending before *T*.

Table 3 groups by the related measures of an information walk *p*, the feature vector *γ* of a candidate that of the ground truth *f*, the feature vector *β* of the last node *c* on an information walk. *d*(*c*_*i*_,*j*) refers to profile similarity between two physicians. Each group contains several representatives of the full list explained in our past works (An et al. 2018a, b). We picked the above measures as they boosted predictive performance in other applications (e.g., the result of medical treatment along an information walk (An et al. 2018a)). To mitigate concerns about reverse-causality and to avoid the possible problem of predicting a variable with input features in the future, when we extract features of an information walk in some year (e.g., 2010), we use node centrality measures derived from the network in the previous year (e.g., 2009).

### Baseline methods

In addition to our proposed BPR-IW, the models/metrics below also generate a preference score *X* between a candidate node *j* and the last node *c*, so they could sort their available candidate nodes for a top-*K* subset as the prediction result.

**Most popular (MP).** *X*(*c*,*j*)=*e*(*c*,*j*) It takes the edge weight in history between *c* and a directly connected neighbor. It could be the number of referrals between two physicians. However, the range of candidates is limited.

The performance of traditional link prediction methods are used as benchmarks against which to compare the new methods. Such methods include **Common neighbors (CN) (**Lorrain and White 1971**), Preferential attachment index (PA) (**Liben-Nowell and Kleinberg 2007**), Adamic-Adar index (**Adamic and Adar 2003**)** and **Jaccard index (**Jaccard 1901). Notably, these similarity metrics do not incorporate the other nodes on the observed part of an information walk, and are only applicable for the neighbors that interacted with node *c* before. However, our BPR-IW model extends the range of possibly predicted candidates, even without a direct edge or common connected nodes with the last node *c*.

**Markov Chain (MC) (**Rendle et al. 2010**).** *X*(*c*,*j*)=*Prob*(*c*.*next*=*j*|*c*,*c*.*prev*) The two-gram version incorporates the second-to-last node *c*.*prev* so as to compute the frequency of state transition.

**Long short-term memory (LSTM).** Given the corresponding node sequence of an information walk, we treat the features of all nodes (in Table 3) as the time series inputs into a LSTM model (Hochreiter and Schmidhuber 1997). We aim to explore whether the LSTM model could learn the hidden patterns based on the past node-to-node transitions to yield an output tensor that is very close to the ground truth *f*. However, the hit-rate of LSTM is lower than 0.01 under all parameter settings in our experiment. Another paper (Choi et al. 2016) reported a similar level of failure of LSTM when predicting the next medical visit.

**Transition-based Factorization Machines (TFM) (**Pasricha and McAuley 2018**).** The TFM model merges the current item, next item and user into a 1×*n* vector $\overrightarrow {y}$. It defines a preference score according to Eq. 6, in which *d*^**2**^ is the Euclidean distance function, $\overrightarrow {w}$ is a weight vector, $\overrightarrow {v}$ and $\overrightarrow {v}^{'}$ represent latent embedding and translation vectors, respectively:

6 $$  X\left(\overrightarrow{y}\right)=w_{0}+\sum_{a=1}^{n}w_{a}y_{a}+\sum_{a=1}^{n}\sum_{b=a+1}^{n}d^{2}\left(\overrightarrow{v}_{a}+\overrightarrow{v}^{'}_{a}, \overrightarrow{v}_{b}\right)y_{a}y_{b}.  $$

The hit-rate (defined in Eq. 7) of TFM on the TDI referral data is less than 0.01 under all experimental settings, including an overall $\overrightarrow {y}$ with our proposed network measures and a comparatively plain $\overrightarrow {y}$ with three IDs only (walker, current and next node). The majority of the nodes in the network of physicians have a small node degree (< 4). Therefore, in such a cold-start environment TFM may not perform as well as that on a dense dataset (Pasricha and McAuley 2018) consisting of frequent users and a part of nodes. Meanwhile, when most of the applied network measures are not categorical, TFM does not make full use of its advantage of dealing with the features in one-hot encoding. TFM enumerates all possible pairs of feature interactions, but some of them may not boost the prediction. As a highlight of TFM, it is better for the latent transition vector $\overrightarrow {v}^{'}$ to depend on the past track (i.e., observed walk).

**BPR-no-IW.** *X*(*c*,*j*)=*wd*(*c*,*j*). As a comparative method to BPR-IW, this model only takes the item of physician profile similarity in Eq. 1 to show the power of the other network science measures about an information walk and the related nodes.

As our main purpose is to prove the significance of network science measures for information walk prediction, we leave further development of Factorization Machine (Rendle 2010) -based preference models to future work.

### Results

For a pair consisting of walk *i* and its next node *f*_*i*_ as the ground truth, BPR-IW or some baseline model will return a sorted list of *K* candidate nodes *R*_*i*_. Here we choose two evaluation metrics: hit-rate (HR) and mean percentile rank (MPR) defined by Eq. 7. HR reflects the possibility of presenting the ground truth *f* to users in the returned list, while MPR corresponds to the expected efforts a user may take to find the ground truth.

7 $$  \begin{array}{lcl} HR = \frac{1}{|P_{test}|}\sum_{i \in P_{test}}1(f_{i} \in R_{i}) \\ MPR = \frac{1}{HR\times|P_{test}|} \sum_{i \in P_{test},\ f_{i}\in R_{i}}\frac{rank(f_{i})}{K} \end{array}  $$

**Fig. 3:**
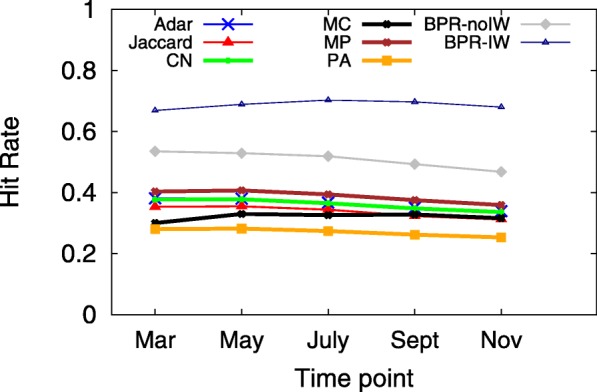
HR at several time points in 2011, when *K*=20

**Fig. 4:**
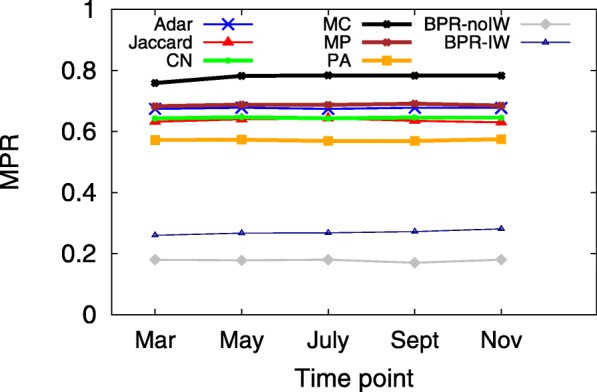
MPR at several time points in 2011, when *K*=20

**Fig. 5:**
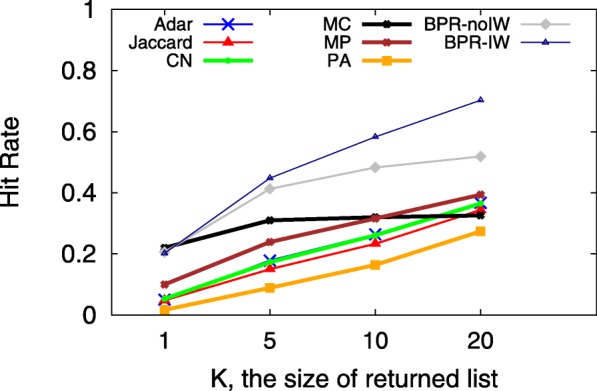
HR with different K on 07/01/2011

**Fig. 6:**
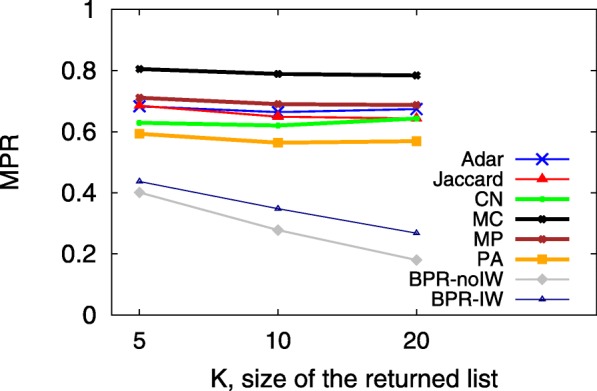
MPR with different K on 07/01/2011

**Fig. 7:**
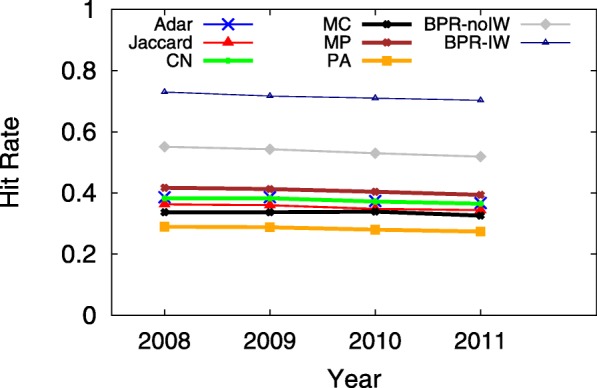
HR on 07/01 during 2008-2011, when *K*=20

**Fig. 8:**
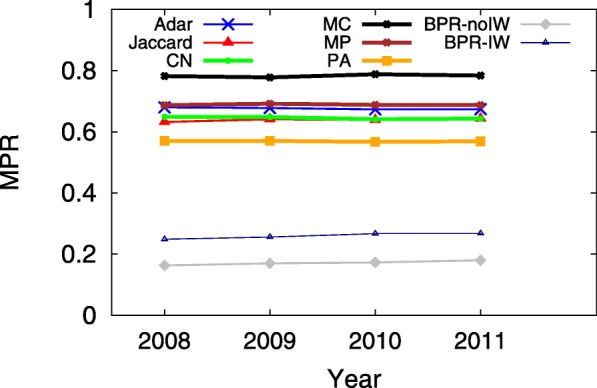
MPR on 07/01 during 2008-2011, when *K*=20

A smaller MPR yet larger HR implies a more accurate predictive model, which indicates that users would see the ground truth on top of the user interface from sorting the returned candidates in decreasing order according to their preference scores. Since the hit-rate values of LSTM and TFM are less than 0.01, Figs. 3, 4, 5, 6, 7 and 8 only present the result of the other successful models. As for the parameters in training process, the *λ*_*Θ*_ in Eq. 2 is 0.001 and the step size in the SGD updating process is 0.05.

Figures 3 and 4 show the HR and MPR at several time points in 2011 for BPR-IW and other baselines, under the setting of *K*=20 in the returned list. In terms of HR, BPR-IW beats the others and BPR-no-IW performs the second best. The other baseline methods get close hit-rate values between 0.3 and 0.4. In addition, BPR-IW and BPR-noIW get the smallest MPR, which suggests the ground truth *f* would be located near the top of the returned list. For most of the models, the different observation time points do not result in obvious gaps in HR or MPR.

Figures 5 and 6 show the impact of *K* on HR and MPR on the same day of observation. Note that in Fig. 6, MPR will be 1.0 for all models if the only returned candidate (*K*=1) hits the ground truth. When *K* increases from 1 to 20, most of the models predict the next node better because the HR increases as well. For our proposed BPR-IW model, under the setting of *K*=20, the HR is over 0.7 for the test set *P*_*test*_ with 10K+ information walks. For MPR, non-BPR models are almost stable when *K* increases, but BPR-IW and BPR-IW display a decreasing MPR from 0.4 to 0.2. As a result, it may be more desirable to choose a slightly larger *K* for BPR related models so that the walk prediction system could present more possible candidates to users, including the key node of ground truth *f*. We compare those models with different *K* values since it is relevant to user experience and needs to be accounted for in the design of a real application, like the number of pages returned on a webpage in response to a search query.

Figures 7 and 8 show the HR and MPR on 07/01 from 2008 to 2011, respectively. It seems that all models perform very stable on the same day in those years, which tends to support that the network structure in the years of 2008-2011 may be steady as well.

Based on two basic features of an information walk, the length and time range, we implement min-max normalization and classify the test set into five groups based on the percentile. We compute the recall for the walks whose ground truth *f* are successfully predicted by the BPR-IW model. Table 4 shows the recall of five groups of information walks on July 1st, 2011. The stable performance in all five groups under varying size of *R* supports that BPR-IW does not adapt to one group (e.g., a longer information walk) much better than another, at least no obvious difference in terms of information walk length and time range.

**Table 4:** The BPR-IW recall of five groups of information walks with several *K* on 07/01/2011

*K*	0 to 20%	20 to 40%	40 to 60%	60 to 80%	80 to 100%
(a) Length (i.e., number of nodes).					
1	0.208	0.202	0.201	0.196	0.174
5	0.462	0.448	0.459	0.436	0.430
10	0.605	0.588	0.601	0.565	0.564
20	0.720	0.710	0.727	0.692	0.690
(b) Time range (i.e., days of the gap between the first and the last physician visiting).					
1	0.191	0.207	0.194	0.216	0.215
5	0.445	0.449	0.454	0.459	0.474
10	0.595	0.590	0.584	0.598	0.613
20	0.727	0.718	0.698	0.714	0.711

The rules of group division are information walk length and time range

Our initial experiments illustrate that features derived from network science and time series analysis for the nodes on an information walk greatly boost HR at the cost of only a slightly larger MPR. We believe it is more desirable and necessary to present the ground truth node to users than the comparative ranking within the list. Therefore, BPR-IW performs the best in our experimental settings. The classical link structure based metrics do not predict as well as BPR-IW, since they do not consider the feature *p* of the whole observed information walk. In addition, they are able to find candidates from the connected or other nearby nodes only, according to the network in history. The BPR framework does not predict the next node directly with a state transition probability. However, the output of relative ranking is enough for the users who do not want to figure out the quantitative reasons behind the prediction. From the perspective of network research, we greatly recommend the application of network measures and the derived information walk features for further related projects. In addition, metadata also provides important features, since the data-specific features (e.g., physician profile similarity) appear presumably to help with successful prediction in the BPR-no-IW model.

## Patterns in network of information walks

We detect statistically significant (*p*-value less than 0.05) patterns between a pair or among several special information walks defined by some structural relationship. The following patterns are derived from the information walks network in the first quarter of 2011. They may suggest hidden patterns in the healthcare system for domain experts to explain and analyze the effects in further research.

- Citing the notion of path-homotopy from algebraic topology, we focus on a pair of homotopic information walks as two information walks which share the common starting and ending nodes in the physician collaboration network. Because of the existence of two guaranteed common nodes, the homotopic information walks are more closely connected in the network of information networks than a pair of non-homotopic walks. Table 5 shows the comparison, in which all the measures are found to be significantly different by a two-sample t-test.

**Table 5:** Comparison of three edge weights in the network of information walks, between the edges connecting homotopic walks and the others connecting two non-homotopic walks

	Jaccard index	Number of visiting records	Sum of RVU
Homotopic pairs	0.552	24.48	45.00
Non-homotopic pairs	0.234	10.28	19.65

The visiting records and RVU refers to the contribution from the common physicians
- “Lifting” refers to a shortcut of a longer information walk. Assuming a longer information walk contains three consecutive nodes *A*→*X*→*B*, another shorter walk contains *A*→*B*, and the rest of the nodes are the same, we treat the two walks as a pair of lifting walks. In the first quarter of 2011, there are 76K pairs of homotopic walks, and the shorter base walks have an average PageRank value of 1.07×10^−5^ while the longer extended walks have an average PageRank value of 1.20×10^−5^. Meanwhile, when putting the middle node X between A and B in the originating physician collaboration network, we find a significant difference in the resulting PageRank centrality of the nodes. The order is X < A < B.
- Information walk composition exists among three groups of information walks. The first group ends with two nodes *A*→*B*, the second one starts with two nodes *B*→*C*, and the third contains the three nodes *A*→*B*→*C* in the middle of the corresponding physician (node) sequence. Those three groups of information walks have significantly different PageRank values in the network of information walks, which are: the first group 1×10^−5^, the second group 9.8×10^−6^, the third 1.09×10^−5^.

## Simulation test of outlier detection

Since the information walks in our physician collaboration network do not have a gold standard comparator, we evaluate the framework of outlier detection and five distance metrics on a mixed set of the originating observed information walks and the simulated outliers. We exploit the training set at a time interval of observation defined by Fig. 2 to get the neighbor (i.e., directly connected) list of every past node (physician). We then take all the information walks beginning within one month of the focal observation to sample from in order to form mixed set. Taking the observation date as 2010-03-01 as an example, from the IWs beginning in April 2010 we randomly pick up 2500 IWs as the normal cases and the other 2500 IWs to generate outliers. To simulate an outlier, we keep the original starting and ending nodes of an IW but randomly replace all the middle nodes with others from the set of nodes located on a pool of IWs. The analysis period begins in the month following the observation period to provide the pool of IWs for node replacement. In this way, for a general test without a specific definition of an outlier information walk, the replacement operation at least alters the track of the whole information walk to some degree, but retains the basic source and target nodes.

**Fig. 9:**
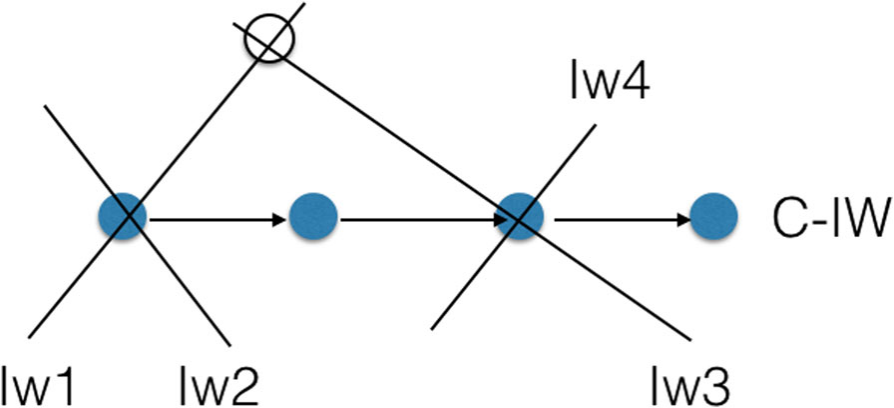
A current information walk (C-IW) consists of four colored nodes. Four different IWs share at least one node with C-IW. Besides, IW1 and IW3 have another common node

**Fig. 10:**
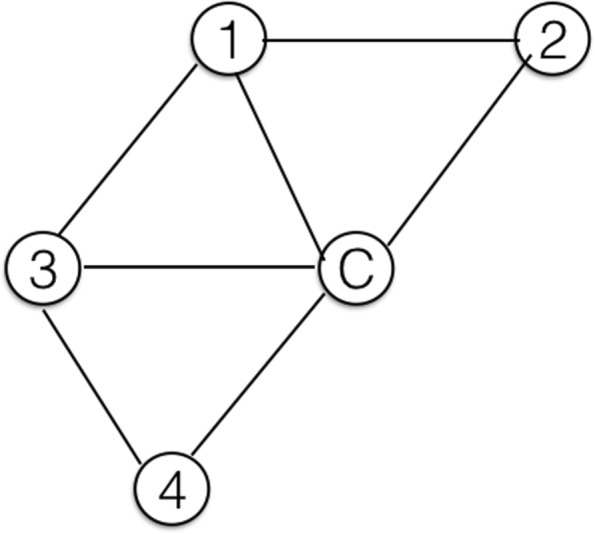
The corresponding network of information walks in Fig. 9

**Fig. 11:**
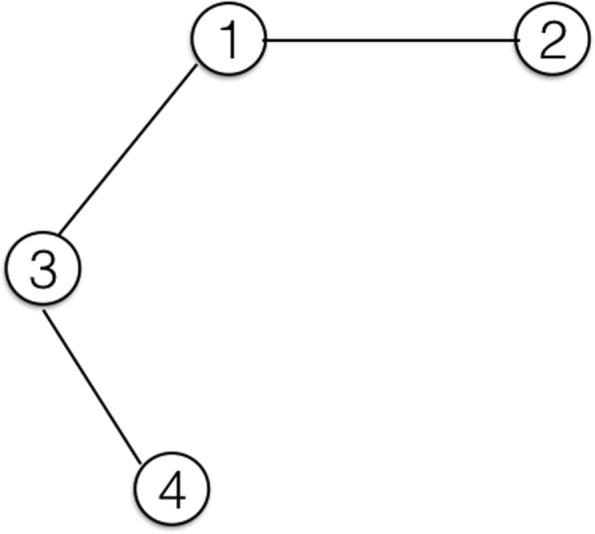
The remaining walk-subnetwork after dropping the current walk (C-IW) from the information walks network in Fig. 10

To apply the five distance metrics between a pair of information walks, we compute the following network science measures for an ongoing/current information walk at each step. They are either popular network measures or special measures to describe the relationship between the ongoing IW and its connected nodes in the network of IWs. Figure 9 gives an example of a current information walk (C-IW) with four connected IWs. Figures 10 and 11 illustrate walk-subnetwork and remaining walk-subnetwork, respectively. The difference between these two local networks shows the alteration of the network itself if the IW is dropped. The comparison metrics are:

1. Number of connected nodes in the network of information walk. Represent the set of nodes (walks) with the ongoing IW as the walk-subnetwork.
2. Number of physicians which are the neighbor of at least one physician on the ongoing information walk.
3. Number of physicians which are the neighbor of at least one physician on a walk in the walk-subnetwork.
4. Average number of covered physicians: the value of measure (3) over that of measure (1).
5. Average Jaccard index weight of those edges within the walk-subnetwork.
6. Network strength of the walk-subnetwork, in terms of the weight of the number of common physicians.
7. Variance of the edge weights in the walk-subnetwork centralization, using the number of common physicians as weights.
8. Transitivity of the walk-subnetwork using the binary undirected edge.
9. Survival rate of edges in the walk-subnetwork if the current IW (i.e. a node) is removed. Denote the left edges and their connected nodes as the remaining walk-subnetwork.
10. Edge density in the remaining walk-subnetwork.
11. Size of the largest connected component in the remaining walk-subnetwork.

**Fig. 12:**
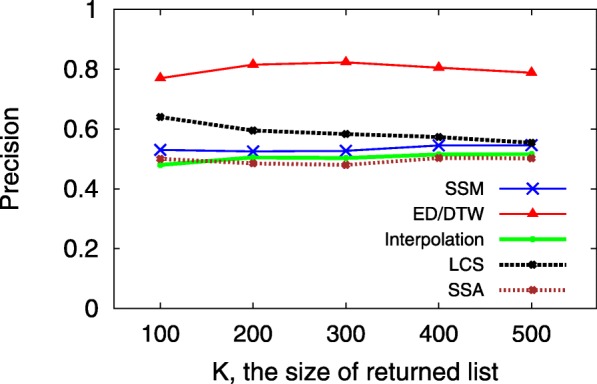
Precision of outlier detection under different Top-K returned walks setting

The evaluation metric is hit-rate (precision), which means the percentage of outliers in the returned K candidates. Figure 12 shows the performance of five distance metrics under their optimal *M* about the choice of a similar neighbor for the outlier score. We tune the neighboring choice parameter *M* for each metric to maximize the hit-rate. Under different values of *K*, ED/DTW performs better than others, and its optimal value is *M*=10. The simulation test is a proof-of-concept of the application of features derived from the network of information walks, which suggests the possibility of the unsupervised proximity based information walk outlier detection. The distance metrics might work better on real outliers. Therefore, to be cautious, we should not judge the best metric based on the current simulation test. Furthermore, we also have multiple options for sampling normal cases and outliers, such as the Bootstrap and the Jacknife (Efron 1992). However, in the simulation test we set a balanced ratio between normal cases and the outliers. The selected feature set of 11 network measures may be expanded and optimized with feature engineering or statistical factor analysis in order to correctly detect an outlier in a new (unseen) dataset.

## Conclusions

In this paper, we exploit the sequence of referrals in a physician collaboration network to solve the problem of next-node prediction on single-track information walks from a network science perspective, explore the network of multiple information walks, and implement a simulation test of information walks outlier detection to support the general idea of an information walk network.

We consider both newly derived information walk features and classical node centrality features to build a BPR-IW model of preference/attraction. The network-based measures yield a flexible BPR-IW model that identifies more possible candidate nodes than the traditional static link prediction method, because in BPR-IW it is not necessary for the last observed node to be directly connected with a candidate. BPR-IW works well on the TDI referral dataset according to a sensitivity analysis which tests both hit-rate and mean percentile ranking across multiple factors, such as the time point (within and cross-year) of observation and the number of nodes in the returned list. BPR-IW could be conveniently applied to other datasets, where network science measures will probably successfully model the structures and relationships among a set of items and nodes.

The network of information walks have several significant patterns (e.g., high clustering coefficient) and provide several features for the simulation test of outlier detection, in which the Edit Distance/Dynamic Time Wrapping based metric performs the best over all metrics in a general proximity based unsupervised framework. Anticipated future work includes the prediction of real outliers defined by domain experts and the subsequent deployment of such an intelligent information walk prediction and detection system.

## Footnotes

1 One will recall that technically a “path" is a sequence of visits of connected nodes with no node visited more than once, while a “walk" only requires the sequence of visited nodes be connected.

2 The Dartmouth Institute for Health Policy and Clinical Practice
